# Toward the Establishment of New Clinical Endpoints for Cystic Fibrosis: The Role of Lung Clearance Index and Cardiopulmonary Exercise Testing

**DOI:** 10.3389/fped.2021.635719

**Published:** 2021-02-25

**Authors:** Elpis Hatziagorou, Asterios Kampouras, Vasiliki Avramidou, Ilektra Toulia, Elisavet-Anna Chrysochoou, Maria Galogavrou, Fotios Kirvassilis, John Tsanakas

**Affiliations:** Pediatric Pulmonology and Cystic Fibrosis Unit, Hippokration Hospital, Aristotle University of Thessaloniki, Thessaloniki, Greece

**Keywords:** lung clearance index (LCI), cardiopulmonary exercise testing (CPET), endpoints, cystic fibrosis, disease severity, disease progression

## Abstract

As Cystic Fibrosis (CF) treatment advances, research evidence has highlighted the value and applicability of Lung Clearance Index and Cardiopulmonary Exercise Testing as endpoints for clinical trials. In the context of these new endpoints for CF trials, we have explored the use of these two test outcomes for routine CF care. In this review we have presented the use of these methods in assessing disease severity, disease progression, and the efficacy of new interventions with considerations for future research.

## Introduction

Cystic Fibrosis (CF) treatment and patient management has improved dramatically over the last 20 years. The rise of new therapies and effective newborn screening has led to an extraordinary increase in overall survival and quality of life. Despite these improvements in clinical practice, the need for methods that can detect early pathological alterations and direct management remains important.

Spirometry is considered the gold standard endpoint for evaluating CF lung disease. Despite its catholic implication for routine CF assessment, it is not feasible from an early age. In children who are able to perform effort dependent lung function tests, spirometry has low sensitivity in detecting early structural and functional alterations (1). This inability to detect early-stage disease results in a “diagnostic gap” (2, 3) that can be crucial to detect if subsequent patient deterioration is to be avoided.

Cystic fibrosis clinicians and researchers have tried to fill this gap with methods that provide early and reliable deterioration assessment. The Multiple Breath Washout (MBW) test with Lung Clearance Index (LCI) and Cardio-Pulmonary Exercise Testing (CPET) have shown promise in detecting early changes in respiratory physiology.

## Lung Clearance Index, LCI

Lung Clearance Index (LCI) is a measure of ventilation distribution inhomogeneity. It can be calculated with the Multiple Breath Washout Method (MBW). The above method measures the clearance of an inert gas (N2 or SF6) from the lungs during tidal breathing. The greater inhomogeneity of ventilation, the higher LCI values are calculated (4). LCI sensitively detects pathology within the peripheral airways (5). This means it can provide information about the “silent lung zone,” the lung compartments whose pathology is not detectable by conventional lung function tests such as spirometry (5, 6). LCI testing is of great value for lung diseases such as CF, for which early detection of structural damages and immediate interventions are crucial to prevent disease progression (7).

MBW is feasible from a young age to adulthood. It requires only passive cooperation with tidal breathing (8, 9). The success rate of the test is 72–99% except for infants and young children below 3.5 years of age, who might need sedation to achieve acceptable measurements. In experienced centers the test can still be used successfully in the majority of these younger children. In experienced centers, over 90% of children aged over 6 year can successfully complete this test and the success rate is even higher amongst adults (10–14).

Kent et al. have reviewed the reliability of LCI and found that the mean coefficient of variation (CV) and the intra-class correlation coefficient (ICC) for LCI measurements within one session were acceptable in both healthy controls and CF cases (15). Few studies have assessed the validity of LCI. Those that have been published demonstrated that LCI was able to distinguish CF subjects from healthy control subjects (16–21). Additionally, LCI could differentiate the disease severity among CF patients with mild to moderate lung disease as revealed by structural changes on high-resolution computed tomography (HRCT) and the patient's microbiological status (3, 7, 22–25). LCI is not well-tolerated by individuals with very severe lung damage, because of the time it takes to complete a measurement. For such individuals, LCI values do not accurately reflect the disease severity because of either totally obstructed or poorly ventilated lung units that do not participate in ventilation distribution. For such patients, spirometry may be a preferable option although this can also plateau at low levels in those with more severe disease (6).

When considering reference values, while LCI was considered an age-independent measure, the current literature suggests a different upper limit of normal (ULN) that is age dependent. The ULN in recent studies has been variably suggested as being; (i) 7.8–8.2 for infants (21, 26–28), (ii) 7.4–7.8 for preschoolers (26, 29), and (iii) 7.2–7.9 for school-aged children (23, 30, 31). Such values might well-depend on the particular equipment used.

### Assessment Tool for Disease Severity

Spirometry has been a routine method for assessing lung function but is insensitive in detecting early airway damage (3, 32). In contrast, HRCT detects structural lung damage even in asymptomatic infants and children with normal spirometry (3, 33). Its use however is limited because of the necessary radiation exposure that occurs with scanning.

The superiority of MBW compared to spirometry regarding the sensitivity in detecting early demonstration of lung disease has been demonstrated in a large number of studies (3, 21, 23, 29, 31, 34, 35). In early stages of CF lung disease, many cases with normal FEV1% had abnormal LCI (3).

LCI also showed high sensitivity (92.3%) and positive predictive value (97.3%) in detecting structural damage as revealed by HRCT. A normal LCI can, in most cases, exclude the presence of HRCT abnormalities (3, 22). LCI has been found to relate in particular to the extent of bronchiectasis, and the presence of mucus plugging and emphysema (36). Such findings suggest that routine use of LCI is a reliable alternative to CT as a first line investigation to exclude lung damage whilst minimizing radiation exposure. Some studies recently investigated the performance of Magnetic Resonance Imaging (MRI) using hyperpolarized gas among patients with CF and also found a strong association between abnormalities detected and elevations in LCI (37–41).

LCI values have been investigated in relation to cough swab and sputum culture results among CF patients (16, 29, 42, 43). Patients with chronic lung infection with *Aspergillus fumigatus, Pseudomonas aeruginosa*, and *Stenotrophomonas maltophilia* had higher LCI levels than those with normal oral flora (44, 45). LCI values were associated with chronic infection with known CF pathogens (16) and pathogen load (42). Spirometry, LCI, and CT measures were all worse in patients colonized with *P. aeruginosa* compared to *Staphylococcus aureus*. It would appear that infection with the latter did not affect the subject's airways as significantly (44, 46, 47).

Ventilation inhomogeneity has been used to assess impacts on the daily life of CF patients. LCI has been shown to be predictive of exercise intolerance as assessed by cardiopulmonary exercise testing (48, 49). Chelabi et al. supported the utility of LCI to predict exercise limitation in children with normal FEV1 (50). Recently, Papale et al. evaluated the ability of LCI to predict nocturnal hypoxemia among CF patients and found that LCI was predictive of such abnormalities (AUC: 0.96, Youden index: 0.79) in stable patients with mild to moderate disease, FEV1 was predictive only in patient with more severe airways disease (AUC: 0.71) (51).

### Assessment Tool for Disease Progression

LCI can be used to assess airway disease in infancy. Higher LCI, measured shortly after birth, correlated with a greater respiratory rate during the first year of life (52). Additionally, LCI values at the age of 3 months were predictive of LCI measurements during the first year of life (53). Normal LCI tended to remain stable (53) whereas those with raised levels tended to track at a higher level (54) despite there being minimal structural changes in this age group (55). Newly diagnosed patients discovered because of clinical symptoms had higher LCI values than those diagnosed through newborn screening (53). Abnormal LCI in preschool children is associated with many clinical parameters including F508del homozygous genotype, *P. aeruginosa* colonization and *nebulised dornase* alpha use. It is also predictive of spirometry in later childhood (56), as well as LCI during school age (57) and adolescence (58).

A significant benefit of LCI has been predicting the risk of first isolation of *P. aeruginosa* in previously non-colonized patients. An increase of LCI by 1.18 could predict first colonization (sensitivity 52%, specificity 70%), despite 81% of such patients being free of respiratory symptoms and an almost stable FEV1% (59). LCI values have also been shown to be predictive of the risk of pulmonary exacerbations with a cut-off of LCI change of 1.37 predicting the likelihood of such clinical events (sensitivity: 47.8%, specificity: 79.6%) (44). Studies suggest that a larger proportion of exacerbation events had a deterioration of LCI compared to a decline in FEV1% (41.7 vs. 30.0%, *p*:0.012) (60). Vermeulen et al. showed that the baseline LCI value of a patient could also predict the risk of developing a pulmonary exacerbation during the following year. Higher baseline LCI values were associated with earlier pulmonary exacerbation (61).

### Assessment Tool of Interventions Efficacy

A remarkable role of LCI is its utility in estimating the effectiveness of therapeutic interventions. LCI improved significantly in CF patients after receiving IV antibiotic treatment for a pulmonary exacerbation. The improvement of LCI was greater than the change in spirometry (25 vs. 15%), but neither LCI nor FEV_1_ recovered to baseline values (60, 62, 63). In contrast there were no significant changes in LCI in studies assessing the effects of nebulized tobramycin (28-day on/off regime) (64, 65). The positive impact of inhalation of dornase alpha (66) and hypertonic Saline (67–69) has been demonstrated using LCI measurements. More recently LCI has been used as a primary outcome measure to assess the efficacy of CFTR potentiators (ivacaftor, lumacaftor/ivacaftor, tezacaftor/ivacaftor) (70–72).

Although the value of LCI has been established in an increasing number of CF services, the main limitations on its more widespread use is that is a time-consuming test requiring specialist equipment and appropriately trained personnel.

### Cardio-Pulmonary Exercise Testing

Cardio-pulmonary exercise testing (CPET) provides a comprehensive assessment of pulmonary, cardiovascular and muscular function. Its principle is quite simple; implementing maximal exercise in a patient while monitoring their cardiac function, pulmonary gas exchange, and progressive muscle oxidation. The test can provide useful data about early pathological alterations in each of these compartments and information about the extent to which these abnormalities impact on exercise performance.

Exercise Testing on a cycle ergometer or treadmill is feasible from the age of around 6 years (73). The test procedure varies in terms of duration depending on the protocol used and the information that need to be obtained (74). The suggested protocol for routine clinical practice does not usually last more than 10–15 min (73, 75) and results are immediately available for interpretation. The test protocol for CF patients has been standardized and reference value equations are available even for young children (76).

CPET's use in CF was mainly research-oriented until, in early 1992, when Nixon et al. demonstrated that peak oxygen uptake during a maximal cardiopulmonary exercise test (the amount of oxygen a CF patient's lung can absorb during maximal exertion) is a prognostic factor for survival. Patients with VO_2_peak >80% predicted showed excellent 8-year survival, whereas patients with low maximal oxygen uptake (VO_2_ peak <60%) had worse outcomes (77).

CPET has since been used for clinical and research purposes in CF with studies highlighting its role in assessing overall disease progression. CPET parameters have also been shown to reflect structural and functional abnormalities and in particular, disease prognosis.

### Assessment Tool of Structural, Functional Abnormalities, and Disease Severity

Cystic fibrosis is characterized by progressive inflammation and impaired mucus excretion (78). This vicious cycle leads to airway remodeling and impaired gas exchange (79). Exercise testing is more sensitive than spirometry in detecting structural abnormalities, as shown by High Resolution Computed Tomography (HRCT) both in adults (80) and CF adolescents (81). CPET is also indicative of functional alterations taking place in CF lungs. CPET parameters have been shown to be associated with ventilation inhomogeneity (48) and reflect increased dead space ventilation in CF patients as the disease progresses (82). Van de Weert et al. found that low exercise capacity was associated with chronic *P. aeruginosa* colonization and changes in immunoglobulin levels in CF adolescents (83).

### Assessment Tool for Disease Progression and Survival

As Cystic Fibrosis lung disease progresses and pseudomonas colonization is established, exercise testing parameters also deteriorate (84). However, the most clinically relevant contribution of CPET to clinical practice is the implications of test results for long term survival. Aerobic fitness as indicated by VO_2_ Max has a key role in determining prognosis (85, 86). In addition other indices including VE/VO_2_ and VE/VCO_2_ have also proved useful as predictors of death or lung transplantation at 10-year follow-up (87, 88).

### Assessment Tool of Intervention Efficacy

Exercise testing has shown additional benefits over spirometry in characterizing for example the efficacy of therapeutic interventions to treat pulmonary exacerbations (89, 90). It has also been used as an outcome measure for assessing the effectiveness of CFTR modulators (87, 91, 92) and as an incentive for as well as measure of the effectiveness of pulmonary rehabilitation (93–96). It has also been used for exercise prescription (97–100), pre- and post- transplantation assessment (101–103) and as a primary and secondary endpoint measure in clinical trials (95, 104, 105).

## Conclusion

LCI and CPET parameters are sensitive measures for detecting early lung disease, and predicting outcomes including colonization with infecting pathogens, pulmonary exacerbations and long term survival, Table 1. These findings have led to the use of these methods for routine patient assessment in many CF centers. Whilst data confirming the sensitivity of LCI and CPET in the early detection of CF lung disease are increasing, there is still a need for more information about validity, age specific reference values and their best use as tools to measure the benefits of clinical interventions. Future research might usefully afford greater insight into the best use of these measures in assessing the needs of increasingly patients with CF.

**Table 1:** The role of LCI and CPET parameters in disease progression, prognosis, and intervention efficacy.

	**LCI**	**CPET parameters**
Disease progression	• Early detection of CF lung disease even though normal spirometry • Sensitivity to demonstrate structural damages shown on CT or MRI • Correlation with pathogens isolated from sputum cultures	• VE/VO2, VE/VCO2 reflect structural damage • VO2peak correlates with ventilation inhomogeneity
Prognosis	• Assessment tool in infancy and preschool age. Predictive for later lung function • Detection of the first isolation of *P. aeruginosa* even without symptoms • A predictor of PEx • Prediction of exercise intolerance and nocturnal hypoxemia	• Overall survival predicted by VO2peak, VE/VCO2 • PEx: • VE/VO2, VO2peak, PETCO2 predictive of future exacerbations
Intervention efficacy	• Assess the effectiveness of IV antibiotics, dornase alpha and hypertonic saline • Primary outcome measure of CFTR modulators	• Reliable outcome measure of interventions (intravenous antibiotics, CFTR modulator therapy, exercise prescription, transplantation assessment) • Richer data than spirometry in assessing therapeutic interventions • Usefulness as a meaningful endpoint for clinical trials

## Author Contributions

EH, AK, VA, IT, E-AC, MG, FK, and JT have made contributions to the design, editing, and writing of this manuscript. All authors contributed to the article and approved the submitted version.

## Conflict of Interest

The authors declare that the research was conducted in the absence of any commercial or financial relationships that could be construed as a potential conflict of interest.
